# Phytophotodermatose à la Mentha rotundifolia

**DOI:** 10.11604/pamj.2014.18.192.4923

**Published:** 2014-07-04

**Authors:** Hind Benhiba, Badredine Hassam

**Affiliations:** 1 Université Mohammed V – Souissi, Service de Dermatologie-Vénéréologie, CHU Ibn Sina, Rabat, Maroc

**Keywords:** Phytophotodermatose, photo-toxicité, Mentha rotundifolia, genou, phytophotodermatitis, phototoxicity, Mentha rotundifolia, knee

## Image en medicine

Les phytophotodermatoses sont des éruptions cutanées engendrées par le contact avec une substance végétale et exagérées lors de l'exposition au rayonnement solaire. Elles sont dues à la présence de furocoumarines (psoralènes, xanthotines, bergaptènes) dans la plante et font intervenir deux mécanismes de photosensibilisation cutanée: photo-toxicité ou photo-allergie. Nous rapportons une observation clinique d'une patiente de 52 ans, ayant comme antécédent des gonalgies chroniques rebelles aux antalgiques et aux anti-inflammatoires habituels, qui a eu recours à un traitement traditionnel comportant un cataplasme contenant de la *« Mentha rotundifolia »*, appliqué pour plusieurs heures sous exposition solaire. L'ouverture du pansement a mis en évidence une brûlure du deuxième degré superficiel des deux genoux, sans d'autres lésions à distance. Le diagnostic de phytophotodermatose a été posé cliniquement. Les soins prodigués à base d'antiseptiques, dermocorticoïdes et crème cicatrisante ont permis la restauration cutanée en quelques semaines. Nous souhaitons à travers cette observation rappeler que le recours à la médecine traditionnelle doit se faire avec précaution. Certes, les cataplasmes aux herbes ont parfois des effets bénéfiques, mais ils ne sont pas dénués de dangers. Leur utilisation doit être prudente en l'absence d’études scientifiques qui prouvent leurs propriétés médicinales. Dans notre observation, l'utilisation à but antalgique a causé des lésions similaires à une brûlure chimique, induisant un clivage dermo-épidermique et formation de vésiculo-bulles. Il s'agit d'une phytophotodermatose jusque là jamais décrite à notre connaissance.

**Figure 1:**
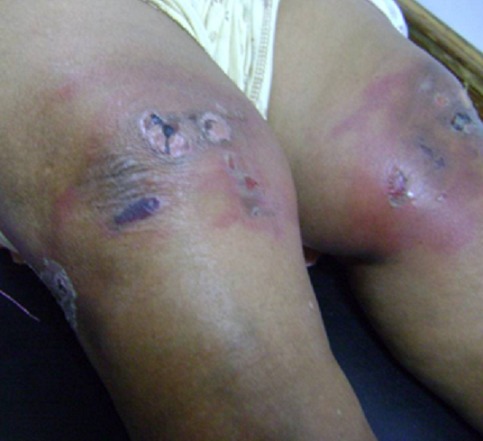
Phytophotodermatose des genoux

